# Mechanism of transient photothermal inactivation of bacteria using a wavelength-tunable nanosecond pulsed laser

**DOI:** 10.1038/s41598-021-01543-5

**Published:** 2021-11-16

**Authors:** Ichiro Tatsuno, Yuna Niimi, Makoto Tomita, Hiroshi Terashima, Tadao Hasegawa, Takahiro Matsumoto

**Affiliations:** 1 Graduate School of Medical Sciences, Nagoya City University, Nagoya, 467-8601 Japan; 2 Department of Physics, Faculty of Science, Shizuoka University, Shizuoka, 422-8529 Japan; 3 Graduate School of Design and Architecture, Nagoya City University, Nagoya, 464-0083 Japan

**Keywords:** Biological techniques, Microbiology

## Abstract

There is a great demand for novel disinfection technologies to inactivate various pathogenic viruses and bacteria. In this situation, ultraviolet (UVC) disinfection technologies seem to be promising because biocontaminated air and surfaces are the major media for disease transmission. However, UVC is strongly absorbed by human cells and protein components; therefore, there are concerns about damaging plasma components and causing dermatitis and skin cancer. To avoid these concerns, in this study, we demonstrate that the efficient inactivation of bacteria is achieved by visible pulsed light irradiation. The principle of inactivation is based on transient photothermal heating. First, we provide experimental confirmation that extremely high temperatures above 1000 K can be achieved by pulsed laser irradiation. Evidence of this high temperature is directly confirmed by melting gold nanoparticles (GNPs). Inorganic GNPs are used because of their well-established thermophysical properties. Second, we show inactivation behaviour by pulsed laser irradiation. This inactivation behaviour cannot be explained by a simple optical absorption effect. We experimentally and theoretically clarify this inactivation mechanism based on both optical absorption and scattering effects. We find that scattering and absorption play an important role in inactivation because the input irradiation is inherently scattered by the bacteria; therefore, the dose that bacteria feel is reduced. This scattering effect can be clearly shown by a technique that combines stained *Escherichia coli* and site selective irradiation obtained by a wavelength tunable pulsed laser. By measuring Live/Dead fluorescence microscopy images, we show that the inactivation attained by the transient photothermal heating is possible to instantaneously and selectively kill microorganisms such as *Escherichia coli* bacteria. Thus, this method is promising for the site selective inactivation of various pathogenic viruses and bacteria in a safe and simple manner.

## Introduction

As the global prevalence of severe acute respiratory syndrome coronavirus 2 (SARS-CoV-2) outbreaks increases, there is a great demand for developing and demonstrating novel disinfection technologies to protect against various pathogenic viruses and bacteria. The recent pandemic of SARS-CoV-2, the causal agent of COVID-19, is not only contagious through respiratory droplets but can also spread through nasal, oral and eye mucus-contaminated surfaces^1^. Moreover, it has recently been suggested that SARS-CoV-2 could be airborne^2^, although clear evidence for such transmission has not yet been presented. Furthermore, SARS-CoV-2's ability to survive in aerosols for at least 3 h and up to 72 h on plastic surfaces was recently demonstrated^3^, suggesting long-term infection risks.

In this situation, ultraviolet (UV) irradiation offers both an effective and a convenient method^4,5^ for inactivation of pathogenic microorganisms, including coronaviruses^6–10^. UV inactivation can occur via several mechanisms, such as damage to nucleic acids^11–15^, proteins^16–18^, and/or internal production of oxygen radicals^19,20^. With the development of UV light emitting diodes (LEDs), various UV inactivation techniques have been reported for many viruses, including SARS-CoV-2 and bacteriophages^8–10,21–23^.

However, it is also known that some types of viral and bacterial pathogens are resistant to UVC radiation; these include blood-borne pathogens such as human immunodeficiency virus (HIV)^24–26^. Moreover, UVC is strongly absorbed by human cells and protein components; therefore, it raises concerns about damaging plasma components^16^ and causing platelet aggregation^17^. Thus, inactivation with UVC radiation can be unsafe when applied as irradiation to the human body to inactivate pathogenic bacteria, viruses and other microorganisms attached to the skin or inside the human body. To avoid the above problems related to the human body, many alternative methods have been studied, such as inactivation by using cold plasma^27,28^, far-UVC light (200–220 nm region)^6–8,29,30^, and plasmonic effects^31,32^. However, these methods are still based on high-energy photons or plasma jets, and their effects on the human body have not yet been clarified.

Inactivation using femtosecond (fs = 10^–15^ s) lasers has attracted special interest as a potential alternative to UV irradiation^33–35^ because this method is based on low-energy photons in the visible or near-infrared region (400–800 nm). The inactivation mechanism is reported as impulsive stimulated Raman scattering of an ultrashort fs visible/near-infrared (NIR) laser pulse. The fs laser pulse coherently excites the mechanical vibrations^36,37^ of the protein capsid of target viral particles, leading to damage and inactivation of a broad spectrum of viruses and bacteria^33–35^ without using toxic or carcinogenic chemicals. This method seems to result in minimal adverse effects on the human body^38^. However, fs laser inactivation methods have the following disadvantages: (i) fs laser systems are very expensive and are not readily available, (ii) the inactivation efficiency is low; thus, it requires a long treatment time of more than 1 h^34–36^ for inactivation, and (iii) it requires an extremely high peak power of the fs pulse for the inactivation of micrometre-sized bacteria^39^. These features impede the scalability and practical implementation of this photonic inactivation process.

To overcome the drawbacks of the fs laser inactivation methods described above, we have demonstrated the efficient inactivation of dye-infused, micrometre-sized bacteria by using a low-power and easily available nanosecond (ns) visible pulse laser (532 nm) and obtained 3-log inactivation of *Escherichia coli* (*E. coli*) bacteria over a short period of treatment time, i.e., within 20 min^40^. In that study, the inactivation mechanism was analysed based on a simple optical absorption effect, which induces transient photothermal evaporation in *E. coli* bacteria. However, through subsequent research involving an investigation into site-selective irradiation with a tunable pulsed laser, we found complex inactivation behaviour that cannot be explained by the simple optical absorption effect.

In this study, to show the principle of inactivation by transient photothermal heating, we first experimentally demonstrate the increase in the transient temperature of gold nanoparticles (GNPs). We show that extremely high temperatures above the melting point can be easily obtained by exciting the plasmon absorption band with a tunable pulsed laser. The reason we used GNPs for the demonstration is that their thermodynamic and thermophysical properties are well established compared to those of small organic structures such as bacteria and viruses. Here, GNPs of various diameters were photothermally melted by instantaneous pulse laser irradiation, and the transient photothermal behaviour of GNP colloids versus the diameter was elucidated based on both the optical absorption spectrum and electron microscopy images. We consider that the results of our previous study^40^, in which the transient temperature behaviour had been theoretically estimated, are now experimentally confirmed in this work.

Second, based on the GNP results, we demonstrated the efficient inactivation of various strains of *E. coli* by using a tunable pulsed laser. By investigating the wavelength dependence of the inactivation efficacy, we observed complex inactivation behaviour that cannot be explained by the simple optical absorption effect. In this study, we found that the scattering effect^41,42^, which was not previously discussed, plays an important role in inactivation because the input irradiation is inherently scattered by the bacteria; therefore, the dose that bacteria feel is reduced by scattering. To investigate both the scattering and absorption effects for various wavelength regions, we fused visible light absorbing dye with *E. coli*^40^, because the refractive index as well as the extinction coefficient can be modified by this staining treatment. The complex inactivation behaviours can be clearly shown by comparative experiments between unstained and stained *E. coli* with site-selective (various wavelength) irradiation obtained by a wavelength tunable pulsed laser. In contrast to UV inactivation, the inactivation by tunable photothermal heating instantaneously destroys and kills *E. coli* cells, which is confirmed by Live/Dead fluorescence microscopy measurements. The combination of tunable pulse laser irradiation and chromophore-fused target bacteria and/or viruses result in the selectivity of the inactivation, which cannot be obtained by UV inactivation methods. Hence, this simple method selectively inactivates a desired virus or bacterium over other various microorganisms.

## Materials and methods

### Culturing, staining and enumeration of microorganisms

A pure culture of *E. coli* strain O1, ATCC 8739, or DH5α was incubated in nutrient broth (E-MC63; EIKEN Chemical Co., Japan) at 37 °C for 20 h. A concentration of 10^9^ to 10^11^ colony forming units (CFU)/mL was achieved and used for the experiments. To stain *E. coli* with a dye solution, we used crystal violet (CV) dye (Hayashi Pure Chemical Industry Limited Corporation, Japan). The CV dye solution consisted of 1 g of CV dye, 0.9 g of ammonium oxalate, 10 ml of ethanol and 90 ml of pure water. The details of the staining process are described in Ref. 40. It should be noted that the reduction behaviour (ageing) of stained *E. coli* was almost the same as that of unstained *E. coli*; for example, both stained and unstained *E. coli* showed approximately a 10% reduction in CFU after 1 h of experiments; therefore, we consider that the staining process does not affect the viability of *E. coli*. To perform the inactivation experiments by using the tunable pulse laser, 600 μL of the stained bacterial cells was taken. Colonies were counted after incubation for 24 h at 37 °C. Plates yielding 1 to 5,000 CFU were considered for analysis. All experiments were performed at least three times independently. Log inactivation was calculated as log (N/N_0_), where N is the CFU number after irradiation and N_0_ is the CFU number before irradiation.

### Inactivation by tunable nanosecond laser pulse

**Figure 1:**
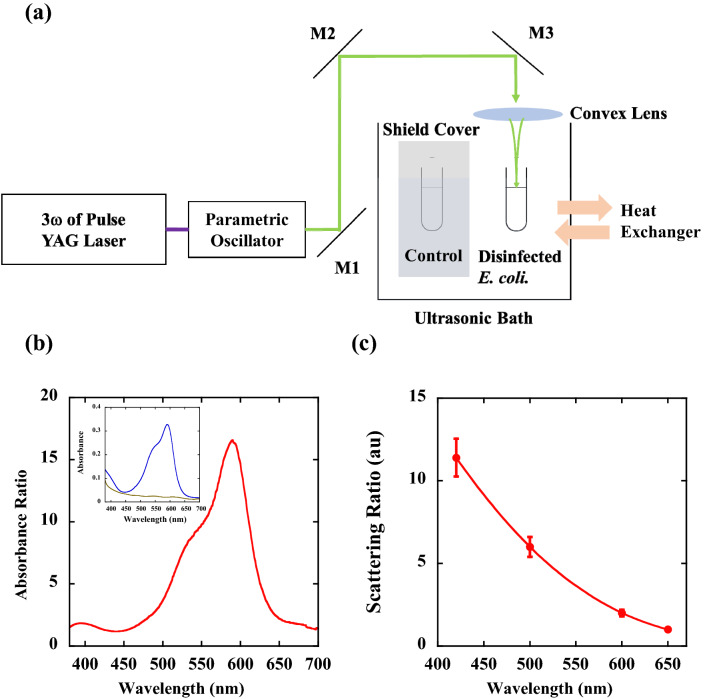
Experimental setup and optical properties. (**a**) Optical setup of the tunable pulse laser inactivation system. (**b**) Optical absorbance ratio (α_cv_/α_0_) between stained (α_cv_) and unstained (α_0_) *E. coli* O1 solutions. The inset shows the optical absorption spectrum (absorbance) of unstained *E. coli* solution (brown line) and a crystal violet dye-stained *E. coli* solution (blue line). (**c**) Relative ratio of scattering intensity as a function of pulse laser wavelength for *E. coli* O1. Here, we normalize the scattering intensity of both stained (σ_cv_) and unstained (σ_0_) *E. coli* O1 to 1 at 650 nm.

Figure 1a shows the optical setup for the inactivation system. Wavelength tunability was obtained by using an optical parametric oscillator (OPO) (BasiScan 120 HE-KE, Spectra-Physics, USA) pumped by the third harmonics (3ω) of a pulsed YAG laser (GCR-150–10, Spectra-Physics, USA). The OPO emits both signal and idler wavelengths, whose tunability is obtained by a phase-matching condition. We only used the signal beam for inactivation by using dichroic mirrors. The signal beam can be varied in the wavelength range from 400 to 650 nm. The pulse duration of 10 ns, repetition rate of 10 Hz, and pulse energy of 10 mJ were maintained at the same values within these wavelength ranges. A laser beam was guided to a microtube made of borosilicate glass with 5.7 mm φ × 50 mm, which contained a suspension of *E. coli* (600 μL). The focusing beam was made by using a convex lens with a focal length of 100 mm, and the centre of the suspension was irradiated, as shown in Fig. 1a. The power of the pulsed laser beams was maintained at 100 mW, and the corresponding power density was 50 W/cm^2^. The inactivation reaction occurred at the central spot on the glass microtube, where the diameter of the focused beam was approximately 0.5 mm determined from our beam profile measurements (scanning slit method), and the irradiated region was approximately 4 μL (0.5φ × 20 mm). The suspension in the tube was homogeneously diffused by using an ultrasonic bath with a frequency of 46 kHz. The temperature of the ultrasonic bath was maintained at 23 °C by using a heat exchanger, where the heat exchanger played a role in inhibiting a temperature increase due to 60 min of ultrasonic operation. We note here that the temperature of the microtube would have risen to approximately 50 °C throughout the 60 min of ultrasonic operation (not due to laser irradiation) without the heat exchanger. The control suspension, which was not subjected to laser irradiation, was also placed in the ultrasonic bath to precisely distinguish the inactivation caused by the ultrasonic effect from that caused by laser irradiation. However, it should be noted that the CFU reduction by the ultrasonic treatment was less than 10% of the CFU of the initial control sample; therefore, we used the *E. coli* CFU from the suspension in the ultrasonic bath as the control sample.

### Live/Dead assay by fluorescence microscopy

The inactivation mechanism by pulsed laser irradiation is not clear; whether this method gives death to *E. coli* or just disappearance of cultivability. The living or dead states of *E. coli* before and after pulsed laser irradiation was determined by Live/Dead assay using a fluorescence microscopy^43–45^. Here we use BD™ cell viability kit (BD Bioscience, CA, United States) as per the manufacturer’s protocol. Briefly, bacterial cells were stained with thiazole orange (TO) and propidium iodide (PI). The fluorescence from live or dead cells was observed using a fluorescence microscope (Olympus, Tokyo, Japan) with a × 40 objective lens. The excitation of the TO was performed by an excitation light from a high-pressure mercury lamp transmitted through a band-pass filter between 460 and 490 nm. The fluorescence of the TO was measured through a 515 nm long-pass filter (named as “green fluorescence channel”). On the other hand, the excitation of the PI was performed by the same light transmitted through a band-pass filter between 520 and 550 nm and the fluorescence of the PI was measured through a 580 nm long-pass filter (named as “red fluorescence channel”). We evaluate the living bacteria with or without cultivability by the signal from the green fluorescence channel and the dead states by the signal from the red fluorescence channel. To show that the living bacteria with and without cultivability emit the same green fluorescence, we performed deep ultraviolet (DUV) inactivation experiments for *E. coli* DH5α. The green fluorescence images and the colony plates (cultivability) are compared without (control) or with DUV irradiation (λ = 265 nm and dosage of 60 mJ/cm^2^) emitted from a DUV-light emitting diode (265-FL-02-G01, Dowa Electronics Corp. Tokyo, Japan).

## Results

### Transient temperature increase of Au nanoparticles by pulsed laser excitation

It is difficult to directly measure the temperature of small organic structures such as viruses and bacteria by pulsed laser beam irradiation. However, the quantitative transient photothermal behaviours can be clearly shown by using inorganic nanoparticles because their thermodynamic and thermophysical properties are well established. Here, we show that extremely high temperatures above the melting point of GNPs can be achieved by pulsed laser irradiation. We also show that continuous wave (CW) laser irradiation with the same wavelength and dose as those of the pulsed laser does not increase the temperature of GNPs. This evidence is directly confirmed by optical absorption spectra and scanning electron microscopy (SEM) images.

**Figure 2:**
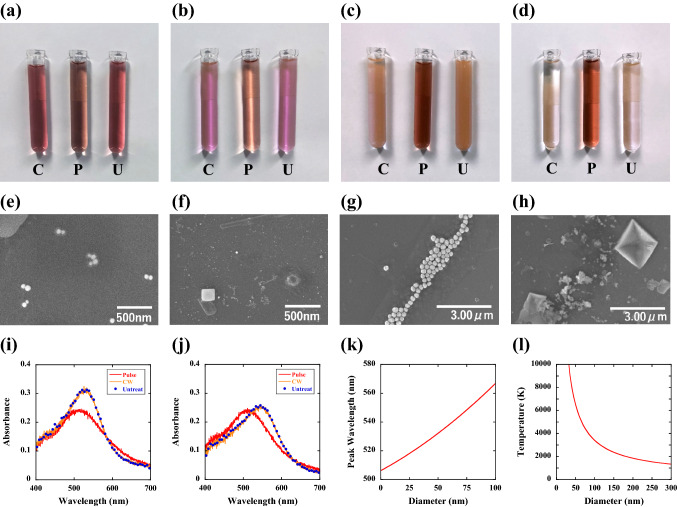
Physical state observations of gold nanoparticles by pulse laser irradiation. (**a–d**) Colour change of Au nanocolloid suspensions by pulsed laser irradiation for diameters of (**a**) 40 nm, (**b**) 80 nm, (**c**) 150 nm, and (**d**) 300 nm. Here, C denotes the suspension after continuous wave laser irradiation, P denotes the suspension after pulsed laser irradiation, and U denotes the suspension before laser irradiation (control). (**e**–**h**) Scanning electron microscopy images of the Au nanoparticles before and after pulse irradiation. (**e**) 40 nm nanoparticles before irradiation, (**f**) 40 nm nanoparticles after irradiation, (**g**) 300 nm nanoparticles before irradiation, and (**h**) 300 nm nanoparticles after irradiation. (**i**-**j**) Optical absorption spectra for (**i**) 40 nm nanoparticles and (**j**) 80 nm nanoparticles. Here, the red line shows the spectrum treated by pulsed laser irradiation, the orange line is the spectrum treated by CW laser irradiation, and blue dots are the spectrum of untreated samples. (**k**) Experimentally determined relation between the diameter of Au nanoparticles and the peak value of the absorbance spectrum. (**l**) Theoretically calculated maximum temperatures of Au nanoparticles versus diameter. The temperature of the Au nanoparticles smaller than 300 nm exceeds the melting point (1337 K); thus, all the Au nanoparticles from 40 to 300 nm are instantaneously melted by pulsed laser irradiation.

Figure 2a–d show the colour change of GNP colloid suspensions by pulsed laser irradiation. We used various sizes of colloid suspensions (G-40–20: 40 nm and 7.15 × 10^10^/ml, G-80–20: 80 nm and 7.82 × 10^9^/ml, G-150–20: 150 nm and 3.60 × 10^9^/ml, G-300–20: 300 nm and 4.50 × 10^8^/ml) in 0.1 mM phosphate buffered saline solutions produced by Cytodiagnostics Inc. in Canada. The colloid suspension was placed in the disinfected *E. coli* area denoted in Fig. 1a. The diameters of the GNPs were 40 nm (Fig. 2a), 80 nm (Fig. 2b), 150 nm (Fig. 2c), and 300 nm (Fig. 2d). Here, we compare the photothermal reaction behaviours for both CW laser beam (denoted by C), pulsed laser beam (denoted by P) irradiation, and untreated samples (denoted by U). The power of both the CW and pulse laser beams was maintained at 100 mW, and the corresponding power density was 50 W/cm^2^. The excitation wavelength was set to 532 nm to coincide with the absorption band of the surface plasmon resonance^46–48^. The duration of irradiation was 30 min. Interestingly, although both the CW and the pulsed laser beams had the same irradiation power density and the same wavelength, the CW laser treatments did not change the colour of the colloid suspensions. However, the pulsed laser beam treatments significantly changed the suspension colour for all the diameter sizes of nanoparticles.

Figure 2e–h show SEM (Hitachi S-4800) images of GNPs with 40 nm (e: untreated, f: pulsed laser treated) and 300 nm diameters (g: untreated, h: pulsed laser treated) to confirm the effect of pulsed laser beam irradiation. For the untreated samples (e and g), GNPs of uniform size (size dispersity is less than 10% for both diameters) are dispersed in the sample plate. However, in the treated samples (f and h), we cannot observe GNPs but observe much smaller Au fragments less than 10 nm, which is smaller than the resolution of our SEM system. Furthermore, very large Au crystallites, which were not present before the treatment, can be observed in the SEM images (f: 200 nm-, h: 2 μm-crystallites). The face direction of the Au crystallites is clearly seen in the SEM images. These results show that GNPs absorb the laser pulse and then instantaneously reach a temperature above the melting point (increase of the local temperature), while the CW laser treatments cannot change the local temperature. The dissolution of GNPs leads to smaller Au nanofragments less than 10 nm or to larger Au crystals (the volume of the crystal is more than 100 times larger than that of the original nanoparticles).

To estimate the size of the Au nanofragments, we performed optical absorption spectrum measurements. We note that all the optical absorption spectrum measurements described here were performed by using an integrated sphere (Labsphere Inc. 3P-GPS-020-SL, USA). Figure 2i,j show the absorbance spectra for 40 nm- (Fig. 2i) and 80 nm- (Fig. 2j) GNPs, respectively. Here, the red lines represent the spectra treated by pulsed laser irradiation, the orange lines represent the spectra treated by CW laser irradiation, and the blue dots represent the spectra of untreated samples. For the CW laser treatments, the absorbance spectra show the same curves as those of the untreated samples; this result indicates that the temperature increases of 40 nm- and 80 nm-GNPs are small; therefore, the nanostructures of both Au colloids remain unchanged. However, in the pulsed laser treatments, the peaks of the absorbance for both 40 nm- and 80 nm-GNPs shift to shorter wavelength regions below 510 nm. Figure 2k shows the experimentally determined relationship between the diameter of GNPs and the peak value of the absorbance spectrum. This relationship suggests that the particle size of the pulsed laser-treated sample is on the order of 10 nm in diameter, which agrees with the SEM observations. Therefore, the absorbance measurements and the colour changes of the Au colloid also suggest that all the GNPs are instantaneously melted by pulsed laser irradiation, and then smaller Au nanofragments less than 10 nm are formed in the solution.

We have previously evaluated the maximum temperatures of GNPs as a function of diameter and absorbed laser fluence (mJ/cm^2^)^40^. The transient response of the temperature (local temperature) as a function of time, *t*, can be given as:

1 $$\left( {T_{L} - T_{0} } \right) = \frac{\theta S}{{\rho c\nu }}E_{p} \exp \left( {{{ - \gamma St} \mathord{\left/ {\vphantom {{ - \gamma St} {\rho c\nu }}} \right. \kern-\nulldelimiterspace} {\rho c\nu }}} \right).$$

On the other hand, the temperature increase of the whole solution system (bulk temperature) can be obtained by averaging Eq. (1) over time, *t*, as:

2 $$\left( {T_{B} - T_{0} } \right) = \frac{\theta }{\gamma }I_{0} .$$

Here, *ρ* is the density (g/cm^3^), *c* is the specific heat (J/g·K), *v* is the volume (cm^3^), *T*_*L*_ is the time-dependent local temperature of GNPs due to the absorption of the pulsed laser radiation (K), *T*_*B*_ is the equilibrium bulk temperature of the solution by the pulsed laser radiation (K), *T*_*0*_ is the temperature before irradiation (K), *θ* is the absorption ratio of laser radiation (dimensionless), *S* is the surface (cm^2^), *γ* is the convective heat transfer coefficient (J/s·cm^2^·K), and *E*_*p*_ is the pulse energy (J/cm^2^). Here, the temporal behaviour of the temperature in Eq. (1) was derived from the heat transfer equation^40^. The maximum temperatures of the GNPs with various diameters are obtained by substituting *t* = 0 into Eq. (1).

Figure 2l shows the theoretically calculated maximum temperatures versus the diameter of GNPs. To obtain this line, we used the density of GNPs of *ρ* = 19.3 g/cm^3^^49^ and a specific heat of *c* = 0.13 J/g·K^49^, *θ* = 2.6 × 10^–3^^40^, and a pulse energy of *E*_*p*_ = 5 J/cm^2^. The theoretically calculated maximum temperatures obtained here coincide with the previously reported values of temperature^49–53^. This curve clearly shows that GNPs smaller than 300 nm (diameter) reach temperatures above the melting point (1337 K); thus, the GNPs of all sizes (40 nm-300 nm) are instantaneously melted by pulsed laser irradiation, leading to the formation of much smaller Au nanofragments. On the other hand, the equilibrium bulk temperature of the solution (*T*_*B*_) after 30 min of pulsed or CW laser irradiation was measured at approximately the same temperature as that of before irradiation (*T*_*B*_-*T*_*0*_ = 3 K). We can conclude that pulse laser irradiation is possible to instantaneously attain the high temperature beyond the melting point of GNPs as shown in Fig. 2l, which is completely impossible by CW laser irradiation.

### Inactivation of unstained *E. coli* by pulsed laser treatments: wavelength dependence

**Figure 3:**
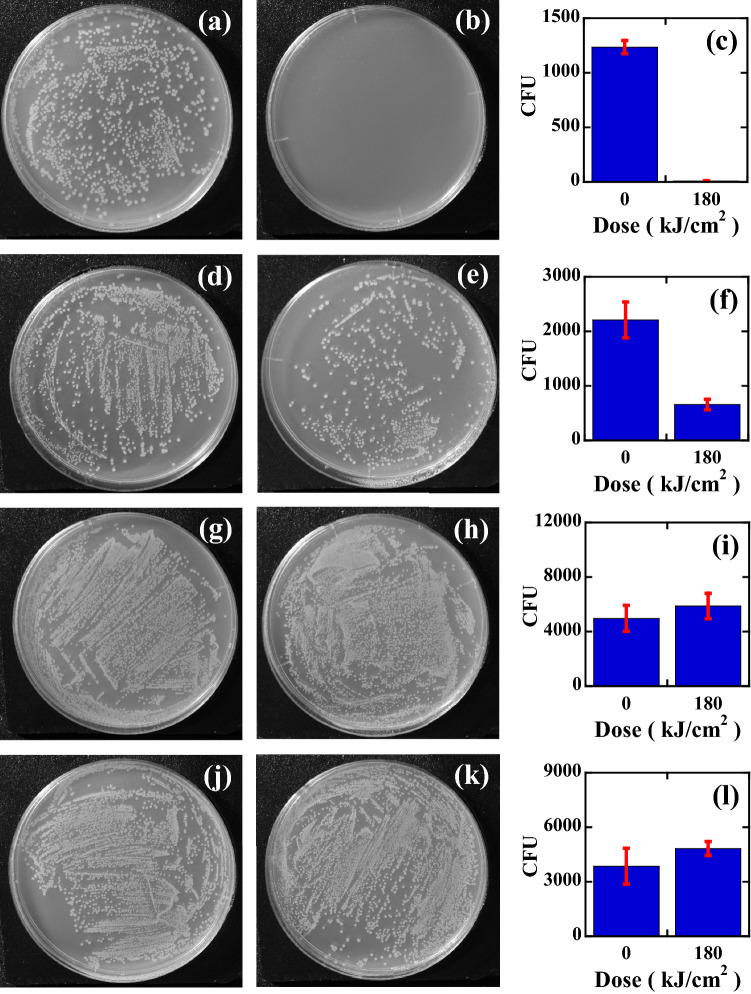
The results of the efficacy of inactivation for unstained *E. coli* O1 by using the tunable pulsed laser. (**a**) Control plate of a 420 nm pulsed laser, (**b**) plate inactivated by a 420 nm pulsed laser, and (**c**) the number of CFU on the control plate (1235 ± 61 CFU) and treated plate (0.33 ± 0.1 CFU) with a 420 nm pulsed laser. (**d**) Control plate of a 500 nm laser, (**e**) plate inactivated by a 500 nm laser, and (**f**) the number of CFU on the control plate (2211 ± 164 CFU) and plate (659 ± 97 CFU) treated with a 500 nm pulsed laser. (**g**) Control plate of a 600 nm laser, (**h**) inactivated plate by a 600 nm laser, and (**i**) the number of CFU on the control plate (4974 ± 953 CFU) and plate (5873 ± 930 CFU) treated with a 600 nm pulsed laser. (**j**) Control plate of a 650 nm laser, (**k**) inactivated plate by a 650 nm laser, and (**l**) the number of CFU on the control plate (3852 ± 986 CFU) and treated plate (4827 ± 383 CFU) with a 650 nm pulsed laser.

The results of the efficacy of inactivation by using a wavelength-tunable pulsed laser for unstained *E. coli* O1 are shown in Fig. 3a–l, where Fig. 3a–c are the results obtained at 420 nm, Fig. 3d–f are the results obtained at 500 nm, Fig. 3g–i are the results obtained at 600 nm, and Fig. 3j–l are the results obtained at 650 nm. The photographs in the left column (a, d, g, j) are the control plates, those in the middle column (b, e, h, k) are the inactivated plates subjected to 180 kJ/cm^2^ dose (50 W/cm^2^ and 1 h irradiation), and the bar graphs in the right column (c, f, i, l) are the number of CFU on the control plates (left bar graph) and the treated plates (right bar graph). With decreasing inactivation wavelength, the efficacy of inactivation increases. For example, the number of colonies was 1235 ± 61 CFU for the control plate and 0.33 ± 0.1 CFU for that subjected to the 180 kJ/cm^2^ dose of 420 nm-inactivation, 2211 ± 164 CFU for the control plate, 659 ± 97 CFU for that subjected to the 180 kJ/cm^2^ dose of 500 nm-inactivation, 4974 ± 953 CFU for the control plate, 5873 ± 930 CFU for that subjected to the 180 kJ/cm^2^ dose of 600 nm-inactivation, and 3852 ± 986 CFU for the control plate, 4827 ± 383 CFU for that subjected to the 180 kJ/cm^2^ dose of 650 nm-inactivation. We plotted the wavelength-dependent CFU response, inactivated CFU by pulsed laser irradiation N(D) divided by the control CFU (N_0_), as shown in Fig. 5a. Here, D is the magnitude of the dose (kJ/cm^2^), N_0_ is the number of CFU in the unirradiated control (CFU/mL), N(D) is the number of CFU at a given irradiation dose D, the blue circles represent the inactivation rates obtained at 420 nm, the green circles represent those obtained at 500 nm, the orange circles represent those obtained at 600 nm, and the red circles represent those obtained at 650 nm. At a glance, the CFU reduction behaviour seems to be correlated with the absorbance spectrum of unstained *E. coli,* as shown by the brown line in the inset of Fig. 1b, where the absorbance of unstained *E. coli* becomes larger for shorter wavelengths. However, it is also found that the reduction rate obtained above is not simply proportional to the absorbance; for example, between 420 and 600 nm, the absorbance differs only 2 times (0.04 at 420 nm and 0.02 at 600 nm); however, the reduction rate differs more than 50 times (0.02 at 420 nm and 0.0003 at 600 nm). Here we note that the pH of the bacterial solution before and after the inactivation does not change and it was about 5.2, which was measured by a pH meter (LAQUAtwin pH-33B, HORIBA Ltd. Kyoto, Japan). In the next section, we will clarify the effect of absorbance on the reduction rates by staining *E. coli*.

### Inactivation of stained *E. coli* by pulsed laser treatments: wavelength dependence

**Figure 4:**
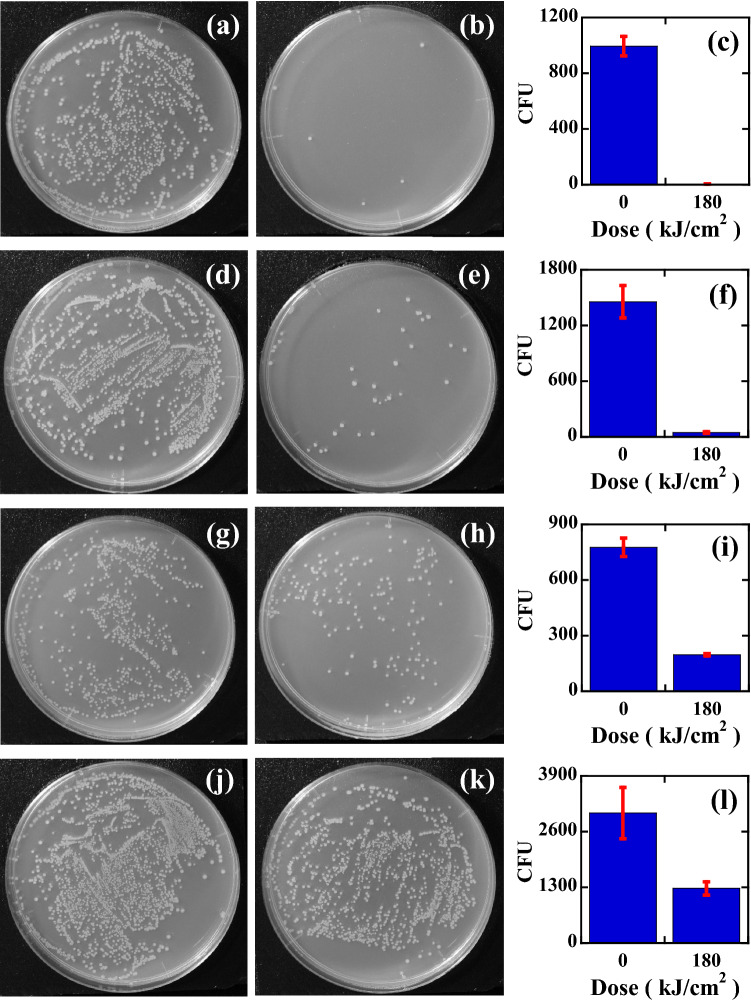
The results of the efficacy of inactivation for crystal violet-stained *E. coli* O1 by using the tunable pulsed laser. (**a**) Control plate of a 420 nm pulsed laser, (**b**) plate inactivated by a 420 nm pulsed laser, and (**c**) the number of CFU on the control plate (995 ± 70 CFU) and treated plate (4.3 ± 0.9 CFU) with a 420 nm pulsed laser. (**d**) Control plate of a 500 nm laser, (**e**) inactivated plate by a 500 nm laser, and (**f**) the number of CFU on the control plate (1457 ± 175 CFU) and treated plate (48 ± 8.5 CFU) with a 500 nm pulsed laser. (**g**) Control plate of a 600 nm laser, (**h**) plate inactivated by a 600 nm laser, and (**i**) the number of CFU on the control plate (776 ± 50.0 CFU) and treated plate (197 ± 6.0 CFU) with a 600 nm pulsed laser. (**j**) Control plate of a 650 nm laser, (**k**) plate inactivated by a 650 nm laser, and (**l**) the number of CFU on the control plate (3034 ± 601 CFU) and plate (1276 ± 158 CFU) treated with a 650 nm pulsed laser.

The results of the efficacy of inactivation by using the wavelength-tunable pulsed laser for *E. coli* O1 stained with crystal violet (CV) dye are shown in Fig. 4, where Fig. 4a–c are the results obtained at 420 nm, Fig. 4d–f are the results obtained at 500 nm, Fig. 4g–i are the results obtained at 600 nm, and Fig. 4j–l are the results obtained at 650 nm, respectively. The photographs in the left column (a, d, g, j) are the control plates, those in the middle column (b, e, h, k) are the inactivated plates subjected to 180 kJ/cm^2^ dose (50 W/cm^2^ and 1 h irradiation), and the bar graphs in the right column (c, f, i, l) are the number of CFU on the control plates (left bar graph) and the treated plates (right bar graph).

**Figure 5:**
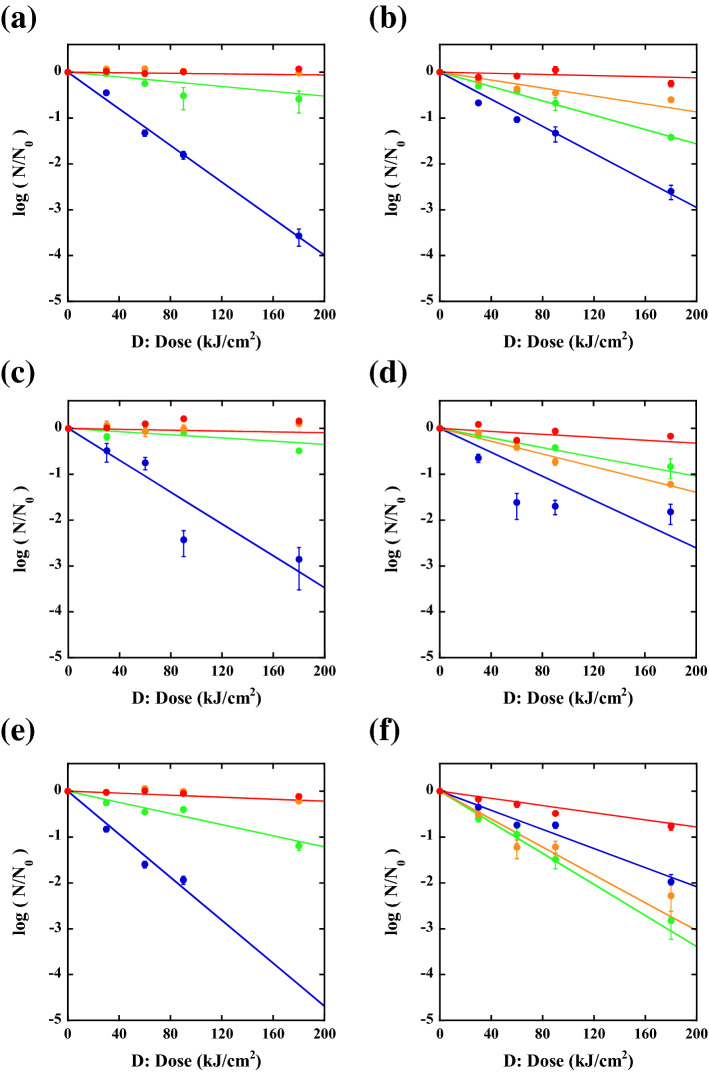
Dose (D) response of stained or unstained *E. coli* inactivated by tunable pulsed laser treatment. The blue circles are the inactivation rates with 420 nm treatments, the green circles are the inactivation rates with 500 nm treatments, the orange circles are the inactivation rates with 600 nm treatments, and the red circles are the inactivation rates with 650 nm treatments. (**a**) Dose response of unstained *E. coli* O1, (**b**) dose response of crystal violet-stained *E. coli* O1, (**c**) dose response of unstained *E. coli* ATCC 8739, (**d**) dose response of crystal violet-stained *E. coli* ATCC 8739, (**e**) dose response of unstained *E. coli* DH5α, and (**f**) dose response of crystal violet-stained *E. coli* DH5α. The dose-based inactivation rate constants Γ (cm^2^/kJ) of stained samples (solid lines of **b**,**d**,**f**) were theoretically obtained based on those of unstained results (solid lines of **a**,**c**,**e**). The experimentally (**a**,**c**,**e**) and theoretically (**b**,**d**,**f**) determined values of Γ (cm^2^/kJ) are described in Table 1.

The absorbance spectrum of the CV-stained *E. coli* is shown by the blue line in the inset of Fig. 1b. Based on the reduction behaviour of the unstained *E. coli*, we expected a correlation between the efficacy of inactivation and the magnitude of the absorbance. However, we found that the obtained results cannot be explained by the simple absorbance model. For example, the number of colonies was 995 ± 70 CFU for the control plate and 4.3 ± 0.9 CFU for that subjected to the 180 kJ/cm^2^ dose of 420 nm inactivation, 1457 ± 175 CFU for the control plate, 48 ± 8.5 CFU for that subjected to the 180 kJ/cm^2^ dose of 500 nm inactivation, 776 ± 50.0 CFU for the control plate, 197 ± 6.0 CFU for that subjected to the 180 kJ/cm^2^ dose of 600 nm inactivation, 3034 ± 601 CFU for the control plate, and 1276 ± 158 CFU for that subjected to the 180 kJ/cm^2^ dose of 650 nm inactivation. We plotted the wavelength-dependent CFU response, by dividing the CFU inactivated by pulse laser irradiation N(D) by the control CFU (N_0_), as shown in Fig. 5b, where the blue circles are the inactivation rates obtained at 420 nm, the green circles are those obtained at 500 nm, the orange circles are those obtained at 600 nm, and the red circles are those obtained at 650 nm.

By comparing Fig. 5a,b, we found the following results: (i) despite the increase in the absorbance, the efficacy of inactivation is reduced at 420 nm irradiation, (ii) approximately one to two orders of enhancement in inactivation by the staining treatment can be observed at 500 nm and 600 nm irradiation, and (iii) in the 650 nm wavelength region, the increase in the absorbance (2.2 times) occurs by the staining treatment; however, the efficacy of the inactivation remains almost the same between unstained- and stained *E. coli* O1. We found that the same results of the above (i), (ii), and (iii) points were obtained by changing the strains of *E. coli*. Figure 5c,d show the dose response of unstained and stained *E. coli* ATCC 8739, and Fig. 5e,f show the dose response of unstained and stained *E. coli* DH5α, where the blue circles are the inactivation rates obtained at 420 nm, the green circles are those obtained at 500 nm, the orange circles are those obtained at 600 nm, and the red circles are those obtained at 650 nm. These complex inactivation behaviours between unstained *E. coli* and stained *E. coli* cannot be explained by the simple optical absorption effect.

## Discussion

We found that the above complex inactivation behaviours can be clarified by considering a scattering effect^41,42^ for both unstained and stained *E. coli*. Figure 1c shows the relative ratio of scattering intensity as a function of pulse laser wavelength. Here, the scattering intensity ratio is defined as the ratio of the scattering intensity of stained *E. coli* to that of unstained *E. coli*. It is generally known that the dose-based inactivation rate constant Γ(λ) (cm^2^/kJ) is linearly proportional to the absorption coefficient as Γ(λ) = κα(λ))^54–56^ where κ is a proportional constant. We consider that the dose D (kJ/cm^2^) is modified by the scattering due to the change of refractive index and extinction coefficient by staining^57,58^. In this case, the dose that bacteria feel is different for stained or unstained *E. coli*. By removing the scattering effect from the dose D, the modified dose D’ can be expressed as D’ = (1-ησ)D, where σ is the scattering ratio and η is the fitting parameter. By considering both the optical absorption and the scattering effects for D’Γ(λ), the dose-based inactivation rate constant of stained Γ(λ)_CV_ (cm^2^/kJ) can be related to that of unstained Γ(λ) (cm^2^/kJ) by:

3 $$\Gamma (\lambda )_{CV} = \frac{{\alpha_{CV} }}{{\alpha_{0} }}\frac{{\left( {1 - \eta \sigma_{CV} } \right)}}{{\left( {1 - \eta \sigma_{0} } \right)}}\Gamma (\lambda )$$

where α_0_ is the absorbance of unstained *E. coli*, α_CV_ is the absorbance of stained *E. coli* and the ratio of absorbance α_CV_/α_0_ is shown in Fig. 1b, σ_CV_ is the scattering ratio of stained *E. coli* (the ratio is shown in Fig. 1c), σ_0_ is the scattering ratio of unstained *E. coli* and we set the value as σ_0_ = 1.0 for all the fittings, and η is the fitting parameter at each strain.

By putting the rate constants of unstained Γ(λ) experimentally obtained (Γ_O1_, Γ_AT_, and Γ_DH5α_) and both the rates of absorption and scattering shown in Fig. 1b,c into Eq. (3), we can theoretically estimate the rate constants of stained *E. coli* Γ(λ)_CV_ (Γ_O1CV_, Γ_ATCV_, and Γ_DH5αCV_) for each wavelength. Here, we use η as 0.041 for *E. coli* O1, 0.041 for *E. coli* ATCC 8739, and 0.053 for *E. coli* DH5α. The experimentally-determined rate constants of the unstained Γ(λ) and the stained *E. coli* Γ(λ)_CV_ (the values in the bracket) and those that were theoretically estimated are summarized in Table 1. The theoretically fitted reduction lines of the stained *E. coli* strains shown in Fig. 5b,d, and f explain the observed wavelength dependence of the inactivation rate at each dose. This agreement suggests the validity of Eq. (3), which shows that for inactivation, we need to consider not only the optical absorption effects but also the optical scattering effects.

**Table 1:** Inactivation rate constants versus irradiation wavelength for unstained and stained *E. coli* strains.

λ (nm)	Γ_O1_	Γ_O1CV_	Γ_AT_	Γ_ATCV_	Γ_DH5α_	Γ_DH5αCV_
420	0.020	0.015 (0.015)	0.017	0.013 (0.013)	0.023	0.012 (0.011)
500	0.0026	0.008 (0.008)	0.0017	0.005 (0.005)	0.0061	0.016 (0.017)
600	0.0003	0.004 (0.004)	0.0005	0.007 (0.007)	0.0011	0.015 (0.016)
650	0.0003	0.001 (0.001)	0.0005	0.002 (0.002)	0.0011	0.004 (0.004)

The rate constants of unstained samples (Γ_O1_, Γ_AT_, and Γ_DH5α_) were determined by experimentally obtained CFU, whereas the rate constants of stained samples (Γ_O1CV_, Γ_ATCV_, and Γ_DH5αCV_) were theoretically determined by using Eq. (3). Here, we denote the experimentally determined rate constants for stained *E. coli* strains in the parentheses as a comparison.

Based on a previous study^40^, the inactivation rate constant was correlated to the maximum temperature (*T*-*T*_0_)_M_ as (*T*-*T*_0_)_M_ = Γ(λ)ξ, where ξ (kJ·K/cm^2^) is the parameter characterized by the pulse energy, and we use ξ = 12.7 × 10^3^ (kJ·K/cm^2^)^40^ at a pulse energy of *E*_*p*_ = 5 J/cm^2^. By using this relation, the maximum temperatures [(*T*-*T*_0_)_M_] of both the unstained and stained *E. coli* strains were calculated. These results are summarized in Table 2. We note here that the temperature shown in the table is described by the absolute temperature (K). It is interesting that the inactivation behaviour (rate constant) of unstained or stained *E. coli* strains is correlated to the maximum temperature and that there might be some threshold for inactivation around the boiling point of water. At present, it is not clear whether the differences in fitting parameters η, especially the differences between *E. coli* O1 (η = 0.041), *E. coli* ATCC 8739 (η = 0.041), and *E. coli* DH5α (η = 0.053), reflect the nature of the *E. coli* strains.

**Table 2:** Theoretically estimated maximum temperatures of unstained and stained *E. coli* strains versus irradiation wavelength.

λ (nm)	T_O1_	T_O1CV_	T_AT_	T_ATCV_	T_DH5α_	T_DH5αCV_
420	884	732	808	694	986	605
500	376	529	351	440	478	719
600	309	414	314	503	332	694
650	309	325	314	325	332	427

Here, the temperatures were denoted in units of absolute temperature (K).

As well as the inactivation rate constants Γ(λ) as a function of irradiation wavelength, we have preliminary investigated the inactivation rate constant Γ(E_p_) as a function of a pulse power density. As is theoretically evaluated in Eq. (1), the rate constant Γ(E_p_) is experimentally shown to be proportional to the pulse power density (not shown in this paper). The quantitative results between the inactivation rate and the pulse power density are now under investigation and will be reported elsewhere.

**Figure 6:**
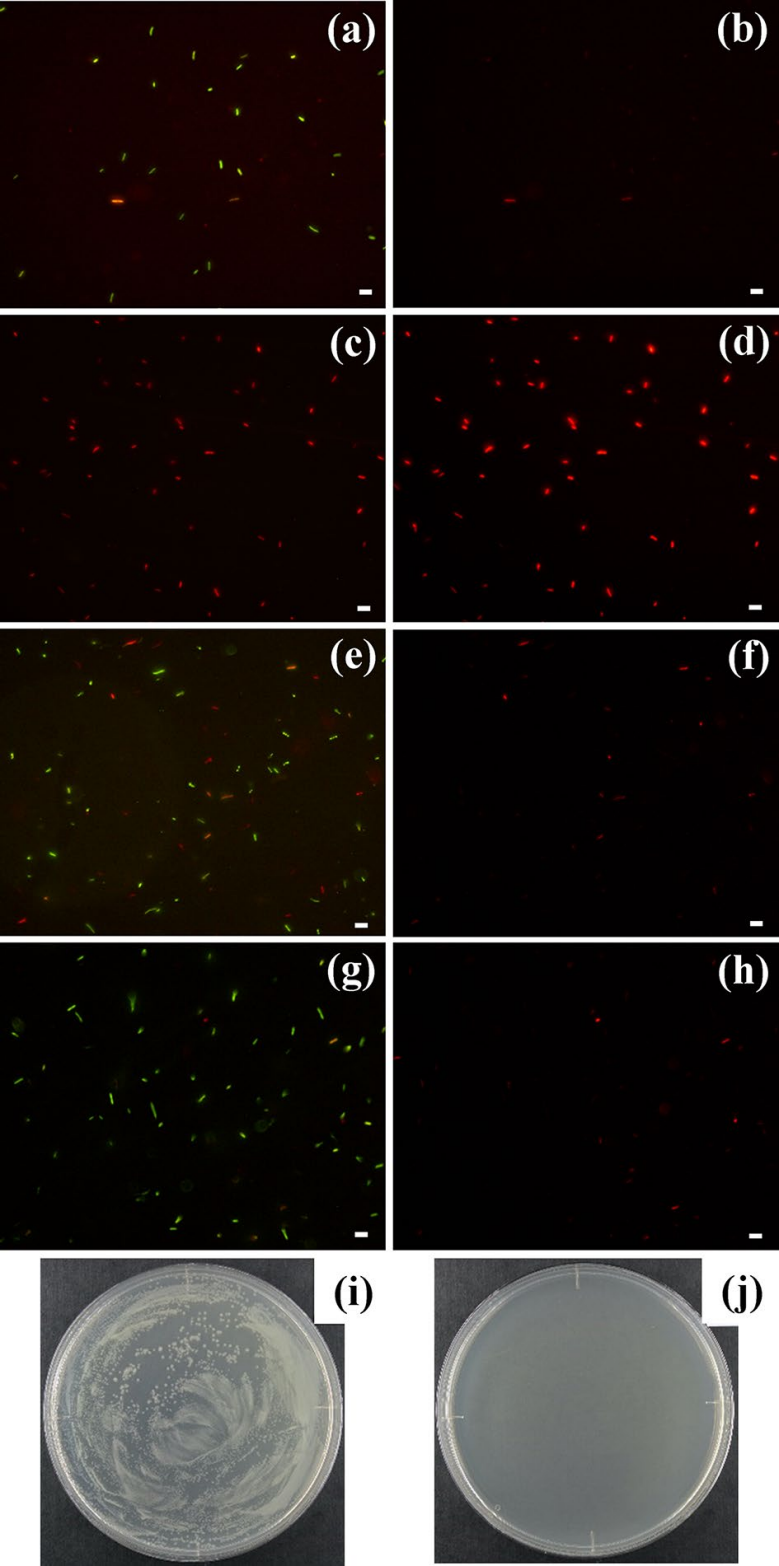
Living or dead states of *E. coli* determined from Live/Dead assay with fluorescence microscopy measurements (10 μm scale bar). (**a**) Green and red, and (**b**) only red fluorescence images before pulsed laser irradiation, and (**c**) green and red, and (**d**) only red fluorescence images after pulsed laser irradiation (420 nm and 180 kJ/cm^2^). (**e**) Green and red, and (**f**) only red fluorescence images before DUV irradiation, and (**g**) green and red, and (**h**) only red fluorescence images after DUV irradiation (λ = 265 nm and doseage = 60 mJ/cm^2^). Images (**a**), (**c**), (**e**), and (**g**) show fluorescence emitted from both thiazole orange and propidium iodide and images (**b**), (**d**), (**f**), and (**h**) show fluorescence emitted from only propidium iodide. Photographs of CFU-evaluations (**i**) before and (**j**) after DUV irradiation are shown to compare with the fluorescence images (**e**–**h**).

We have used laser pulse irradiation to inactivate *E. coli* and have evaluated the efficacy by counting the CFU. However, the method of colony counting cannot determine whether *E. coli* has been dead or has simply lost cultivability. Here, the living or dead states of *E. coli* before and after pulsed laser irradiation was determined from Live/Dead assay using a fluorescence microscopy. Figure 6a–d show the fluorescence microscopy images of Live/Dead cell viability assay of *E. coli* DH5α before and after pulsed laser irradiation (420 nm and 180 kJ/cm^2^). Here, Fig. 6a,c show both of living green fluorescence cells and dead red fluorescence cells, and Fig. 6b,d show only dead red fluorescence cells, respectively. In the control experiment without the pulsed laser irradiation (Fig. 6a,b), most of the cells exhibit green fluorescence. On the other hand, most of the cells with pulsed laser irradiation exhibit red fluorescence (Fig. 6c,d). These results show that the pulsed laser inactivation thermally destroys and kills *E. coli* DH5α cells. However, no significant destruction of *E. coli* cells such as the deformation of cell shape was observed for pulsed laser irradiation; therefore, we consider that *E. coli* was destroyed by local thermal heating effects. Based on the above results of the CFUs and Live/Dead assay, it is found that the pulsed laser irradiation method does not only lose the cultivability but also thermally destroys and kills *E. coli* cells.

It is interesting to compare the results of Live/Dead assay experiments obtained by the pulsed laser inactivation with the inactivation obtained by DUV irradiation. Figure 6e–j show the results of Live/Dead assay using fluorescence microscopy measurements (Fig. 6e–h) and CFU-evaluations (Fig. 6i,j) for *E. coli* DH5α obtained by DUV irradiation (λ = 265 nm and dose = 60 mJ/cm^2^). Because sufficient DUV dosage was given to *E. coli* DH5α, it is considered that DNA damage occurred in almost all *E. coli* DH5α, leading to the loss of the cultivability. Here, Fig. 6e,g show both living green fluorescence cells and dead red fluorescence cells, and Fig. 6f,h show only dead red fluorescence cells, respectively. In the control experiment without DUV irradiation (Fig. 6e,f), most of the cells exhibit green fluorescence. In contrast to the results of pulsed laser-irradiation (Fig. 6c,d), most of the cells exhibit green fluorescence (Fig. 6g,h) despite that the cells are inactivated by DUV irradiation. These fluorescence images are similar to those of non-irradiated control experiment. On the other hand, the results of CFU-evaluations in Fig. 6i (before irradiation) and Fig. 6j (after irradiation) show that the cultivability is completely lost by DUV irradiation. These results provide direct evidence that the DUV inactivation does not kill *E. coli* cells but gives damage to the genes of *E. coli* cells.

Here we note that CV-stained *E. coli* cells were also subjected to the Live/Dead assay using fluorescence microscopy measurements; however, we could not clearly distinguish the live or dead state by the green or red fluorescence intensities. This is because the absorption spectrum of CV overlapped with the green and red fluorescence emission spectra.

## Conclusions

In this study, we first experimentally clarified transient photothermal behaviour by using inorganic GNPs to quantitatively understand the photothermal inactivation mechanism of bacteria or viruses because the thermodynamic and thermophysical properties of GNPs are well established compared to those of small organic structures.

Second, based on the GNP results, we demonstrated the efficient inactivation of various strains of *E. coli* infused with crystal violet dye by using a tunable pulse laser beam emitted from an optical parametric oscillator. By investigating the wavelength dependence of the inactivation efficacy, we found complex inactivation behaviours originating not only from the optical absorption effect but also from the optical scattering effect.

Third, the living or dead states of *E. coli* before and after pulsed laser irradiation was clarified from the Live/Dead assay with fluorescence microscopy measurements. We found that the mechanism of the pulsed laser inactivation originates from the thermal destruction of *E. coli* cells. This is contrary to the results of the DUV inactivation, where the DUV inactivation gives damage to the genes of *E. coli* but does not kill *E. coli* cells. The results obtained here suggest that the combination of tunable pulse laser irradiation and chromophore-fused target bacteria and/or viruses gives the selectivity and flexibility of inactivation.

Furthermore, it is generally known that GNPs strongly bind bacterial lipopolysaccharide (LPS)^59^. Based on this information, it is possible to make Gram negative bacteria that bind with GNPs. The combination of GNPs and tunable pulse laser irradiation could be used to selectively inactivate Gram negative bacteria from a mixed set of Gram-positive and Gram-negative bacteria. We consider that this selectivity can be applied to many fields such as a new inspection method of infection diseases.

There are many bacteria that have inherent absorption bands in the visible region, such as *Pseudomonas aeruginosa* (*P. aeruginosa*), *Staphylococcus aureus* (*S. aureus*), *Micrococcus luteus* (*M. luteus*), and *Kocuria oceani* (*K. oceani*)^60–63^. It is possible to perform site-selective inactivation for these bacteria. We have some promising results of selective inactivation for *M. luteus* and *K. oceani*. These results will be reported elsewhere. We consider that the site selective photothermal heating method proposed here provides disinfection of specific viruses or bacteria from among various microorganisms in a simple and safe manner.
